# Impact of Concurrent Genomic Alterations Detected by Comprehensive Genomic Sequencing on Clinical Outcomes in East-Asian Patients with EGFR-Mutated Lung Adenocarcinoma

**DOI:** 10.1038/s41598-017-18560-y

**Published:** 2018-01-17

**Authors:** Seijiro Sato, Masayuki Nagahashi, Terumoto Koike, Hiroshi Ichikawa, Yoshifumi Shimada, Satoshi Watanabe, Toshiaki Kikuchi, Kazuki Takada, Ryota Nakanishi, Eiji Oki, Tatsuro Okamoto, Kouhei Akazawa, Stephen Lyle, Yiwei Ling, Kazuaki Takabe, Shujiro Okuda, Toshifumi Wakai, Masanori Tsuchida

**Affiliations:** 1 Division of Thoracic and Cardiovascular Surgery, Niigata University Graduate School of Medical and Dental Sciences, Niigata, Japan; 2 Division of Digestive and General Surgery, Niigata University Graduate School of Medical and Dental Sciences, Niigata, Japan; 3 Department of Respiratory Medicine and Infectious Disease, Niigata University Graduate School of Medical and Dental Sciences, Niigata, Japan; 4 Department of Surgery and Science, Graduate School of Medical Sciences, Kyushu University, Fukuoka, Japan; 5 Department of Medical Informatics, Niigata University Medical and Dental Hospital, Niigata, Japan; 6 University of Massachusetts Medical School, Worcester, Massachusetts USA; 7 Division of Bioinformatics, Niigata University Graduate School of Medical and Dental Sciences, Niigata, Japan; 8 Breast Surgery, Department of Surgical Oncology, Roswell Park Cancer Institute, Buffalo, New York USA; 9 Department of Surgery, University at Buffalo Jacobs School of Medicine and Biosciences, the State University of New York, Buffalo, New York USA

## Abstract

Next-generation sequencing (NGS) has enabled comprehensive detection of genomic alterations in lung cancer. Ethnic differences may play a critical role in the efficacy of targeted therapies. The aim of this study was to identify and compare genomic alterations of lung adenocarcinoma between Japanese patients and the Cancer Genome Atlas (TCGA), which majority of patients are from the US. We also aimed to examine prognostic impact of additional genomic alterations in patients harboring *EGFR* mutations. Genomic alterations were determined in Japanese patients with lung adenocarcinoma (N = 100) using NGS-based sequencing of 415 known cancer genes, and correlated with clinical outcome. *EGFR* active mutations, i.e., those involving exon 19 deletion or an L858R point mutation, were seen in 43% of patients. Some differences in driver gene mutation prevalence were observed between the Japanese cohort described in the present study and the TCGA. Japanese cohort had significantly more genomic alterations in cell cycle pathway, i.e., *CDKN2B* and *RB1* than TCGA. Concurrent mutations, in genes such as *CDKN2B* or *RB1*, were associated with worse clinical outcome in patients with *EGFR* active mutations. Our data support the utility of comprehensive sequencing to detect concurrent genomic variations that may affect clinical outcomes in this disease.

## Introduction

Lung tumors are the most prevalent type of cancer and are one of the leading causes of cancer-related death worldwide^1^. The discovery that epidermal growth factor receptor (*EGFR*) mutation is a predictor of clinical response to EGFR tyrosine kinase inhibitors (EGFR-TKIs) has dramatically changed the therapeutic approach to non-small cell lung cancer (NSCLC)^2,3^. In addition to *EGFR*, several oncogenic drivers such as anaplastic lymphoma kinase (*ALK)*, ROS proto-oncogene 1, receptor tyrosine kinase (*ROS1)*, and ret proto-oncogene (*RET)* have been as identified as molecular targets in this disease^4,5^. The effectiveness of molecularly targeted therapies, and advances in technologies for the detection of genomic alterations in tumors, have driven an increasing interest in such precision medicine approaches.

The advent of next-generation sequencing (NGS) has enabled the comprehensive detection of genomic alterations. Utilizing NGS technology, the Cancer Genome Atlas (TCGA) consortium has reported the molecular profiling of 230 lung adenocarcinoma cases, and the detection of mutations in genes such as neurofibromatosis type 1 (*NF1)*, MET proto-oncogene, receptor tyrosine kinase (*MET)*, and erb-b2 receptor tyrosine kinase 2 gene (*ERBB2)*^6^. Although the TCGA represents one of the largest cohorts of NSCLC patients^6,7^, its limitation is that minor ethnicities are not well represented because samples are derived solely from USA-based institutions. It is well known that cancers differ biologically between ethnicities, but to date, genomic data from east-Asian populations have been scarce^8^.

We hypothesized that sequencing a panel of cancer-associated genes using NGS technology would identify essentially all actionable genomic driver mutations in lung adenocarcinoma, regardless of ethnic and geographical background. Moreover, we hypothesized that comprehensive sequencing would identify not only the known actionable driver mutations, such as those affecting *EGFR*, but also other important gene alterations that may impact clinical outcome. To test these hypotheses, we assessed the genomic profile of a Japanese cohort of lung adenocarcinoma patients using NGS-based sequencing of 415 genes, and compared the results with data from TCGA. Finally, it has been reported that the EGFR-mutated patient ratio of east-Asian origin is high compared with Westerners^6,9^, so we evaluated the clinical benefit of this approach by examining the association between concurrent genomic alterations in patients harboring *EGFR* active mutations and their subsequent therapeutic outcome.

## Results

### Patient characteristics

The demographic data for the 100 patients included in this study are shown in Table 1. The median follow-up period after surgery was 32.6 months (range 6.4–104.8). In regard to smoking status, 62 patients identified as never having smoked or as light smokers (pack-years, or PY < 30), and 38 patients were heavy smokers (PY ≥ 30). Overall, the median mutation burden (number of rare SNPs) was 13.5 (range 5–33) and the median number of identified genomic alterations was four (range 1–19), with 98% of patients having one or more actionable mutations, defined as genomic alterations that are either associated with targeted therapy that is FDA/PMDA- approved or would qualify the patient for a clinical trial testing a targeted therapy.

**Table 1:** Patient demographics.

Factor	Category	N = 100
Age (Years)	Median	67
	Range	36–86
Gender (N)	Male	67
	Female	33
Smoking (N)	PY < 30	62
	PY ≥ 30	38
Stage (N)	I	31
	II	24
	III	39
	IV	6

N, number; PY, pack-years.

### The frequency of genomic alterations

Tumors were sequenced with a median coverage of 500× , and a total of 281 individual genomic alterations were identified. *EGFR* was the most commonly mutated gene (48% of patients, n = 48/100), followed by tumor protein p53 gene (*TP53)* (40%) and cyclin-dependent kinase inhibitor 2B (*CDKN2B)* (32%) as shown in Table 2. *EGFR* active mutations, i.e., those involving exon 19 deletion or an L858R point mutation, were detected in 43 patients, with six patients showing more than one mutation in *EGFR* (see Supplementary Tables S1 and S2). Compared with data from TCGA^6,7^, there were significantly more genomic alterations in AT-rich interactive domain-containing protein 1A *(ARID1A)*, *EGFR*, cyclin-dependent kinase inhibitor 1B *(CDKN1B)*, cyclin-dependent kinase inhibitor 2A *(CDKN2A)*, retinoblastoma 1 gene (*RB1)*, phosphatase and tensin homolog (*PTEN)*, activin receptor type 2 (*ACVR2A)*, and F-box/WD repeat-containing protein 7 (*FBXW7)* (all *p* < 0.01) and significantly less genomic alterations in erb-b4 receptor tyrosine kinase 4 gene (*ERBB4)*, Kirsten rat sarcoma viral oncogene homolog gene (*KRAS)*, and Kelch-like ECH-associated protein 1 (*KEAP1)* (*p* = 0.01, *p* < 0.01, and *p* < 0.01, respectively) among our Japanese patients (Table 2).

**Table 2:** Frequency of Gene Alterations in Each Pathway.

	Frequency (%)				Frequency (%)		
	TCGA	This study			TCGA	This study	
	(N = 216)	(N = 100)			(N = 216)	(N = 100)	
Transcription factor/regulator	2.6	0.0	*GATA3*	MAPK signaling	26.3	12.0	***KRAS***
	0.9	0.0	*EP300*		11.8	2.0	***NF1***
	4.0	0.0	*TAF1*		1.8	0.0	*MAP3K1*
	0.4	0.0	*RUNX1*		6.6	5.0	*BRAF*
	3.5	1.0	*WT1*		1.8	0.0	*NRAS*
	0.4	0.0	*FOXA1*		1.3	0.0	*MAP2K4*
	0.4	0.0	*CBFB*	PI(3)K signaling	4.4	6.0	*PIK3CA*
	1.3	0.0	*SOX9*		2.2	17.0	***PTEN***
Histone modifier	6.1	17.0	***ARID1A***		1.3	4.0	*PIK3R1*
	1.8	4.0	*PBRM1*		11.4	0.0	***TLR4***
	7.9	2.0	***SETD2***		5.3	0.0	***PIK3CG***
	4.8	1.0	*KDM5C*		0.0	2.0	*AKT1*
	0.9	0.0	*KDM6A*	TGF-β signaling	3.1	4.0	*SMAD4*
	1.3	0.0	*ASXL1*		0.9	4.0	*TGFBR2*
	2.2	0.0	*EZH2*		2.2	1.0	*ACVR1B*
Genome integrity	51.8	40	*TP53*		0.9	1.0	*SMAD2*
	7.9	3.0	*ATM*		0.9	12.0	***ACVR2A***
	6.1	2.0	*ATRX*	Wnt/β-catenin	9.2	16.0	*APC*
	5.7	10.0	*BRCA2*		3.5	5.0	*CTNNB1*
	5.7	0.0	***ATR***		0.9	0.0	*AXIN2*
	2.6	0.0	*STAG2*	Proteolysis	1.3	13.0	***FBXW7***
	1.3	1.0	*BAP1*		17.1	2.0	***KEAP1***
	3.5	NA	*BRCA1*		0.4	0.0	*SPOP*
	1.3	0.0	*ERCC2*	Splicing	2.2	0.0	*SF3B1*
RTK signaling	11.4	48.0	***EGFR***	HIPPO signaling	1.3	2.0	*CDH1*
	4.0	1.0	*FLT3*	DNA methylation	4.0	0.0	*DNMT3A*
	8.8	2.0	***EPHA3***		3.1	0.0	*TET2*
	7.5	1.0	***ERBB4***	Metabolism	0.9	0.0	*IDH1*
	6.6	2.0	*PDGFRA*		0.4	1.0	*IDH2*
	9.7	0.0	***EPHB6***	NFE2L	2.2	2.0	*NFE2L2*
	3.1	0.0	*FGFR2*	Protein phosphatase	4.6	0.0	***PPP2R1A***
	1.8	3.0	*KIT*	TOR signaling	7.5	1.0	***MTOR***
	0.4	2.0	*FGFR3*		8.8	15.0	*STK11*
Cell cycle	6.6	17.0	***CDKN2A***	Other	3.1	0.0	*NOTCH1*
	5.3	22.0	***RB1***		5.3	0.0	***USP9X***
	3.1	1.0	*CDK12*		0.9	0.0	*NPM1*
	1.8	19.0	***CDKN1B***		10.5	1.0	***HGF***
	0.9	1.0	*CCND1*		1.8	0.0	*AR*
	0.4	0.0	*CDKN1A*				
	0.0	0.0	*CDKN2C*				
	NA	32.0	*CDKN2B*				

Bolded entries represent those genes for which the frequency of alteration was significantly different between TCGA and the present study; N, number; NA, not available.

### Overall impact of genomic alterations on clinical outcome in lung adenocarcinoma

We next assessed the impact of each genomic alteration on the clinical outcomes of patients in our cohort (Table 3). Univariate analysis indicated that patients with *EGFR* active mutations had significantly longer disease-free survival (DFS) than patients without these mutations (*p* = 0.041). Conversely, patients with *TP53* mutation or *CDKN2B* mutation had significantly shorter DFS than patients without those mutations. In multivariate analysis, *TP53* mutation and *CDKN2B* mutation remained independent predictors of DFS (*p* = 0.037 and *p* = 0.002, respectively), but *EGFR* active mutation did not (*p* = 0.050). In regards to overall survival (OS), however, univariate analysis indicated that patients with any *EGFR* mutation, not just those with *EGFR* active mutation, had significantly longer OS (*p* = 0.020 and *p* = 0.027, respectively).

**Table 3:** Univariate and Multivariate Survival Analysis of DFS and OS in All Patients.

Genes	Category	N = 100	5-year DFS (%)	Univariate Analysis		Multivariate Analysis		5-year OS (%)	Univariate Analysis	
				HR (95% CI)	p-value	HR (95% CI)	p-value		HR (95% CI)	p-value
*EGFR* (All)	WT	52	35.5					55.0		
	MUT	48	33.4	0.610 (0.356–1.045)	0.072			77.3	0.356 (0.149–0.847)	**0.020**
*EGFR* (active)^a^	Other^b^	57	31.7			Reference^c^		55.6		
	MUT	43	38.6	0.559 (0.320–0.977)	**0.041**	0.607 (0.335–1.100)	0.050	79.1	0.357 (0.143–0.890)	**0.027**
*TP53*	WT	60	41.5			Reference		76.9		
	MUT	40	28.9	1.795 (1.056–3.051)	**0.031**	1.727 (1.003–2.975)	**0.037**	52.4	2.042 (0.936–4.458)	0.073
*CDKN2B*	WT	68	51.6			Reference		62.9		
	MUT	32	7.1	2.151 (1.253–3.694)	**0.005**	2.391 (1.376–4.155)	**0.002**	70.9	0.761 (0.317–1.823)	0.540
*RB1*	WT	79	36.0					65.4		
	MUT	21	34.3	1.459 (0.783–2.722)	0.234			66.2	1.208 (0.485–3.011)	0.685
*CDKN1B*	WT	81	33.8					61.8		
	MUT	19	40.6	0.948 (0.487–1.844)	0.875			75.9	0.522 (0.179–1.524)	0.234
*CDKN2A*	WT	83	44.4					64.7		
	MUT	17	9.9	1.263 (0.665–2.401)	0.476			64.2	1.071 (0.401–2.860)	0.892
*PTEN*	WT	83	33.3					63.6		
	MUT	17	47.1	1.092 (0.533–2.236)	0.810			72.1	0.880 (0.303–2.559)	0.815
*ARID1A*	WT	83	30.9					63.6		
	MUT	17	58.8	0.811 (0.382–1.722)	0.585			71.7	1.196 (0.449–3.185)	0.721
*APC*	WT	84	37.9					63.8		
	MUT	16	29.5	1.061 (0.533–2.110)	0.866			65.6	1.295 (0.516–3.254)	0.582
*STK11*	WT	85	39.5					67.2		
	MUT	15	18.8	1.707 (0.858–3.395)	0.128			46.7	1.793 (0.672–4.782)	0.243
*FBXW7*	WT	87	30.3					63.6		
	MUT	13	65.9	0.511 (0.203–1.288)	0.155			72.5	1.131 (0.386–3.315)	0.822
*KRAS*	WT	88	37.0					71.9		
	MUT	12	25.0	1.947 (0.950–3.992)	0.069			44.4	2.277 (0.910–5.696)	0.078
*ACVR2A*	WT	88	33.7					63.2		
	MUT	12	56.3	0.493 (0.178–1.366)	0.174			83.3	0.631 (0.149–2.680)	0.533

Note: Only those genes mutated in more than 12 patients were analyzed. ^a^Active EGFR mutation refers to exon19 deletion or L858R point mutations. ^b^Other refers to patients with wildtype EGFR or non-active EGFR mutations. ^c^Multivariate analysis was performed for the EGFR active mutation group, since the data for those with active mutations and those with any EGFR mutation overlapped considerably. DFS, disease-free survival; OS, overall survival; N, number; HR, hazard ratio; CI, confidence interval; WT, wildtype; Mut, mutated; Bold values are those with statistical significance of p < 0.05.

### EGFR genomic alterations and clinical outcome

**Figure 1:**
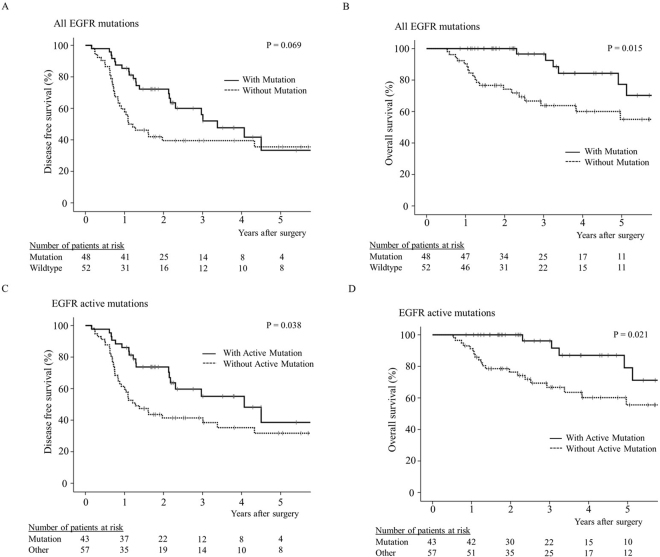
Effect of *EGFR* mutation status on patient survival. Postoperative disease-free survival (**A**) and overall survival **(B**) curves for patients with or without any type of *EGFR* mutation, and disease-free survival (**C**) and overall survival (**D**) curves for patients with or without *EGFR* active mutations (i.e., exon19 deletion or L858R point mutation). ‘Other’ indicates patients with either wildtype *EGFR* or an *EGFR* non-active mutation.

We next examined the clinical outcome of patients with *EGFR* mutations in more detail. The majority of the patients with *EGFR* mutations (84%) identified as either having never smoked or as light smokers (see Supplementary Table S1). In terms of clinical outcome, the 48 patients with *EGFR* mutation showed a trend towards longer DFS and a significantly longer OS, than those without *EGFR* mutation (Fig. 1A,B; log-rank test, *p* = 0.069 and *p* = 0.015, respectively). When only the 43 patients with *EGFR* active mutations were considered, both DFS and OS were found to be significantly longer than for patients with wildtype *EGFR* or *EGFR* non-active mutations (Fig. 1C,D; log-rank test, *p* = 0.038 and *p* = 0.021, respectively).

### Concurrent genomic alterations in patients with EGFR active mutation

**Figure 2:**
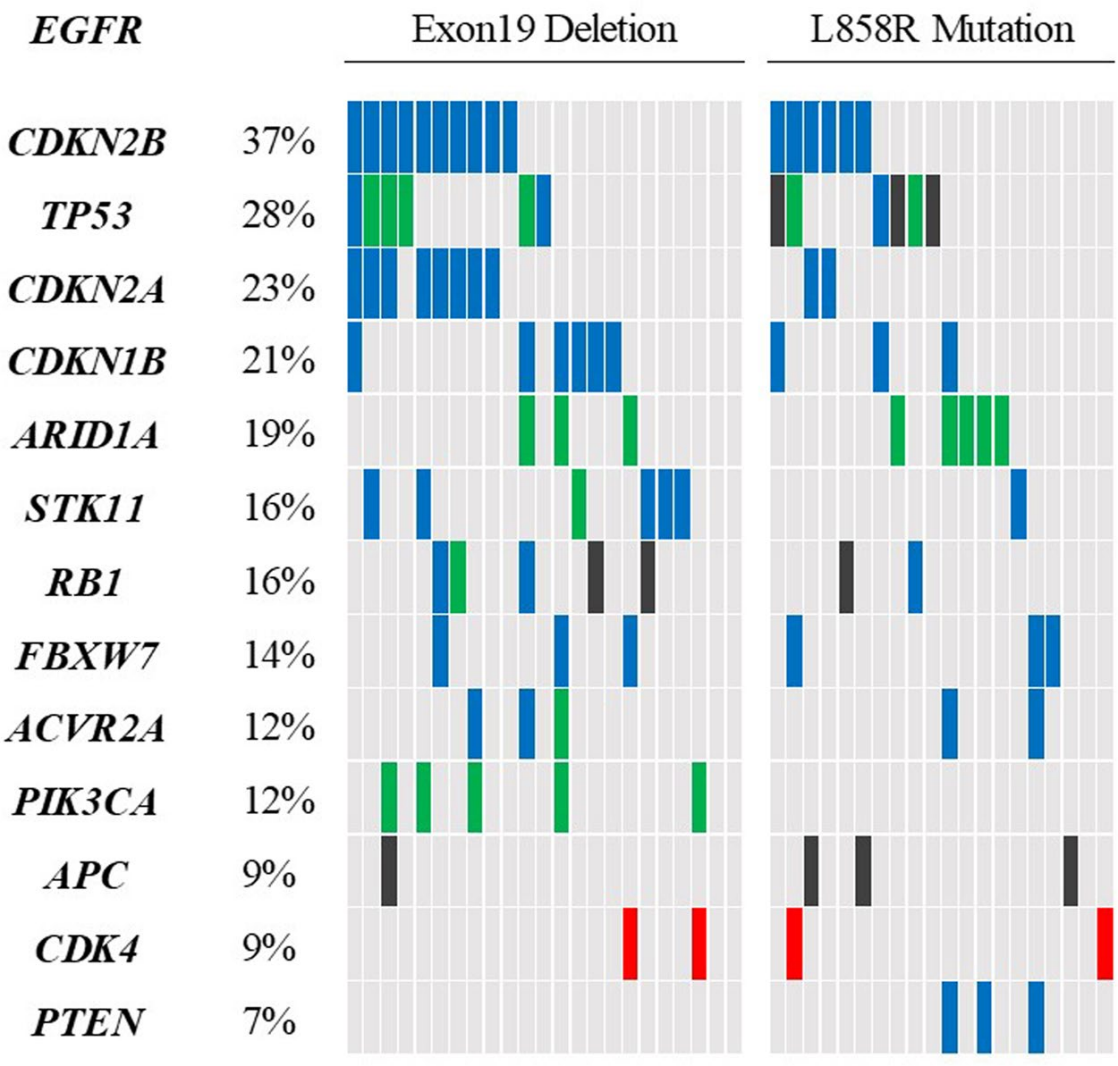
Concurrent genetic alterations among patients with *EGFR* active mutation. Percentages indicate the frequency of each mutation in these 43 patients, with the heatmap indicating the presence or absence of the variation in individuals. Green cells, SNPs; black cells, stop-gain mutations; red cells, gene amplification; blue cells, genomic loss.

Subsequently, we assessed the incidence of concurrent genomic alteration in the 43 patients with *EGFR* active mutation. The most frequent concurrent genomic alterations in these patients were *CDKN2B* (37% of patients), *TP53* (28%), *CDKN2A* (23%), *CDKN1B* (21%), *ARID1A* (19%), *RB1* (16%), and serine/threonine kinase 11 (*STK11)* (16%) (Fig. 2). In agreement with previous reports^10^, there were no concurrent genomic alterations in *KRAS*, *ALK*, *ROS1*, *RET*, B-Raf proto-oncogene (*BRAF)* (V600E) or *MET*. We also examined differences in the pattern of concurrent genomic alteration in patients with *EGFR* active mutation vs. those with either *EGFR* wildtype or *EGFR* non-active mutation. This analysis revealed that patients with *EGFR* active mutation carried fewer *TP53* mutations and *PTEN* mutations than the others (*p* = 0.026 and *p* = 0.018, respectively; see Supplementary Table S3). We also compared the pattern of concurrent genomic alteration in patients with *EGFR* exon19 deletion and those with *EGFR* L858R mutation (Fig. 2); however, no significant differences were found between the two groups.

### Impact of concurrent genomic alterations on clinical outcomes in patients with EGFR active mutation

To assess the impact of concurrent genomic alterations on clinical outcomes in patients with *EGFR* active mutations, survival of these patients was compared to patients with wildtype *EGFR* or *EGFR* non-active mutations. Univariate analysis revealed that patients with *CDKN2B* mutation had significantly shorter DFS than those without *CDKN2B* mutation (*p* = 0.001, Table 4), while patients with *RB1* mutation had significantly shorter OS than those without *RB1* mutation (*p* = 0.035, see Supplementary Table S4). These findings suggest that concurrent genetic alterations affect clinical outcomes in patients with *EGFR* active mutations.

**Table 4:** Univariate Analysis of DFS in Patients with *EGFR* Active Mutations.

Genes	Category	N = 43	5-year DFS (%)	Univariate Analysis	
				HR (95% CI)	p-value
*CDKN2B*	WT	27	51.6		
	MUT	16	7.1	5.618 (1.995–15.827)	**0.001**
*CDKN2A*	WT	33	54.7		
	MUT	10	14.6	1.887 (0.728–4.892)	0.191
*CDKN1B*	WT	34	28.7		
	MUT	9	62.2	0.435 (0.123–1.540)	0.197
*TP53*	WT	31	47.0		
	MUT	12	31.3	2.495 (0.956–6.510)	0.062
*STK11*	WT	36	44.7		
	MUT	7	33.3	0.864 (0.246–3.035)	0.820
*RB1*	WT	36	41.1		
	MUT	7	28.6	1.875 (0.601–5.848)	0.279
*ARID1A*	WT	35	24.1		
	MUT	8	100	0.136 (0.030–2.492)	0.056
*FBXW7*	WT	37	30.3		
	MUT	6	62.5	0.715 (0.196–2.621)	0.613
EGFR*	WT	37	36.5		
	MUT	6	44.4	1.737 (0.566–5.329)	0.334

Note: Only genes mutated in more than six patients were analyzed. *Refers to EGFR non-active mutations, i.e., excluding Exon19 deletion and L858R. DFS, disease-free survival; N, number; HR, hazard ratio; CI, confidence interval; PY, pack-year; WT, wild type; MUT, mutated; Bold values are those with statistical significance of p < 0.05.

We also examined the impact of concurrent genomic alterations on clinical outcomes in patients with wildtype *EGFR* or non-active *EGFR* mutations. In this analysis, patients with *STK11* mutation were found to have significantly shorter DFS (*p* = 0.006, Table 5) and OS (*p* = 0.046, see Supplementary Table S5) than those without *STK11* mutations. Of note, neither *CDKN2B* nor *RB1* were associated with DFS or OS in the patients with wildtype *EGFR* or non-active *EGFR* mutations.

**Table 5:** Univariate Analysis of DFS in Patients with Wildtype *EGFR* or Non-active EGFR Mutations.

Genes	Category	N = 57	5-yr DFS (%)	Univariate Analysis	
				HR (95% CI)	p-value
*TP53*	WT	29	34.4		
	MUT	28	28.3	1.386 (0.720–2.668)	0.329
*CDKN2B*	WT	41	38.9		
	MUT	16	0.0	1.343 (0.667–2.706)	0.409
*RB1*	WT	43	30.0		
	MUT	14	12.8	1.304 (0.612–2.781)	0.491
*PTEN*	WT	43	28.3		
	MUT	14	35.7	1.185 (0.555–2.530)	0.662
*KRAS*	WT	45	33.1		
	MUT	12	25.0	1.571 (0.737–3.348)	0.242
*APC*	WT	45	32.1		
	MUT	12	35.0	0.762 (0.331–1.752)	0.522
*CDKN1B*	WT	47	33.4		
	MUT	10	20.0	1.537 (0.699–3.380)	0.285
*ARID1A*	WT	48	32.4		
	MUT	9	22.2	2.004 (0.871–4.614)	0.102
*STK11*	WT	49	35.3		
	MUT	8	12.5	3.268 (1.402–7.618)	**0.006**
*BRCA2*	WT	49	27.7		
	MUT	8	50.0	0.603 (0.213–1.711)	0.342

Note: Only genes mutated in more than eight patients were analyzed. DFS, disease-free survival; N, number; HR, hazard ratio; CI, confidence interval; WT, wildtype; MUT, mutated; Bold values are those with statistical significance of p < 0.05.

### Clinical outcomes for patients receiving EGFR-TKI therapy

**Figure 3:**
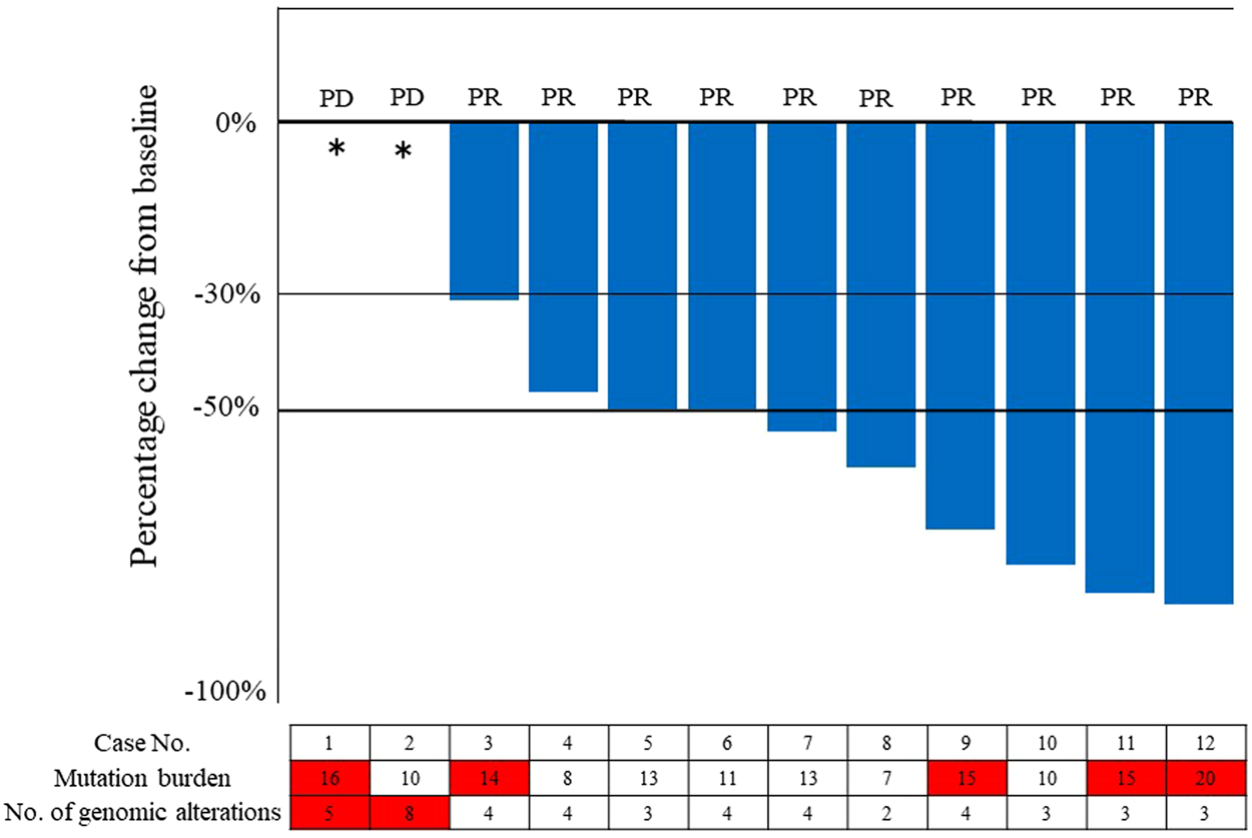
Clinical response of *EGFR* mutated patients treated with EGFR tyrosine kinase inhibitor (EGFR-TKI). The waterfall plot shows the best percentage change in target lesions from baseline for 13 patients treated with EGFR-TKI. Red boxes indicate those patients with a higher than median mutation burden or number of genomic alterations; *indicates patients without a measurable recurrent lesion prior to EGFR-TKI use.

In total, 14 patients received first-generation EGFR-TKI therapy, i.e., gefitinib or erlotinib, for recurrence; however, in one patient an osteosclerotic metastasis in the fourth thoracic vertebra was detected by bone scintigraphy; that lesion was determined to be non-measurable by the RECIST v1.1 guidelines^11^, and thus the patient was excluded from the analysis. One patient received EGFR-TKI therapy, however, the patient was excluded from this study due to serious adverse event happened early after the administration of EGFR-TKI. The others did not develop recurrence or metastatic disease except two: one patient had a recurrence with brain metastasis and received radiation therapy; one patient developed recurrence but had not started treatment yet. Of the remaining 12 patients, two were designated as non-responders (Fig. 3). Interestingly, the number of genomic alterations in the two non-responders was found to be the highest of any of the patients who received EGFR-TKI treatment (Fig. 3).

To gain a clearer understanding of factors that may have contributed to the poor clinical response of these two patients, we examined their medical records in more detail. Briefly, Case 1 initially received right middle lobectomy with lymph node dissection. Two months following surgery, chest CT revealed the presence of mediastinal lymph nodes and supraclavicular lymph node metastases. After the failure of concurrent platinum-based chemotherapy and thoracic radiotherapy, new multiple small (<10 mm) brain and osteosclerotic bone metastases were found to have developed. At this point the patient received EGFR-TKI therapy; however, multiple bone metastases were detected approximately 2 months later. Case 2 underwent right lower lobectomy with lymph node dissection. Seven months after surgery, bone scintigraphy revealed osteosclerotic metastases to the cervical vertebrae. Following irradiation of the cervical vertebrae lesion the patient received EGFR-TKI therapy, but malignant pleural effusion and lymphangiosis carcinomatosa developed approximately 1 month later.

Notably, we investigated the association between the number of genomic alterations and progression-free survival (PFS) of the patients with EGFR-TKI therapy. We found the patients with 4 or more genomic alterations had significantly poorer PFS than those with less than 4 (p = 0.006) (Supplementary Fig. S6).

## Discussion

NGS technologies enable us to identify not only potentially targetable driver mutations but also other important genomic alterations that are associated with clinical outcome. In the present study, we identified actionable genomic driver mutations in 98% of patients in a Japanese lung adenocarcinoma cohort by comprehensive NGS-based sequencing of a panel of 415 genes with relevance for cancer, with an average of 500× depth. In contrast, TCGA performed whole-exome sequencing on tumor, with a mean coverage of 97.6× depth. Although the sequence techniques had been not the same, of note, there were differences in the observed prevalence for several driver gene mutations between the Japanese and TCGA cohorts, consistent with previous reports^12,13^. Furthermore, we found that concurrent mutations, in genes such as *CDKN2B* and *RB1*, may impact the survival of patients with *EGFR* mutations.

The *CDKN2B* gene lies adjacent to the tumor suppressor gene *CDKN2A* in a genomic region that is frequently mutated and/or deleted in various tumor types. *CDKN2B* encodes a cyclin-dependent kinase (CDK) inhibitor known as p15Ink4b, which forms a complex with CDK4 or CDK6 and prevents their ability inactivate the RB1 gene product (Rb) and other Rb-family proteins during the G1 phase of the cell cycle. The p15Ink4b protein thus functions as a cell growth regulator that inhibits cell cycle progression^14^. Zhao *et al*. reported that *CDKN2B* loss is associated with poor overall survival of patients with lung squamous cell carcinoma^15^, and in NSCLC patients, loss of chromosome 9p (encompassing the 9p21.3 locus where *CDKN2A* and *CDKN2B* are located) has also been associated with poor survival outcome^16,17^. Moreover, *CDKN2A* and *CDKN2B* are known to be frequently inactivated by allelic loss and promotor methylation in NSCLC, resulting in the deregulation of cell proliferation through the loss of G1 arrest control^18^.

Like *CDKN2B*, the *RB1* gene is also an important regulator of cell cycle progression. Together with other Rb family members (such as p107 and p130), Rb is phosphorylated by CDK4/6 and other cyclin-CDK complexes, inducing the release of transcription factors of the E2F family and the consequent transcription of genes required for S-phase entry^19^. Molecular alterations involving the CyclinD1-CDK4/6-Rb pathway occur in a variety of malignancies such as breast cancer^20^, prostate cancer^21^, and osteosarcoma^22^, and have been associated with poor prognosis. In regard to lung cancer, recent whole-genome sequencing has revealed that the *RB1* gene is altered in almost all cases of small cell carcinoma^23^. It remains premature to determine the clinical outcome on this population due to the small sample size of this study. However, considering that the CyclinD1-CDK4/6-Rb pathway is downstream of the EGFR signaling pathway, it is likely that this cell cycle pathway, which involves *CDKN2B* and *RB1*, plays an important role in lung cancer progression and/or therapeutic resistance in patients with *EGFR* active mutation.

A previous study has reported that lung adenocarcinoma patients with relapse have a significantly larger proportion of private non-trunk mutations in their primary tumor than those without relapse, indicating not only that subclonal mutations are crucial for tumor progression, but that they increase postoperative risk of relapse^24^. Notably, the two lung adenocarcinoma patients in our cohort that carried the highest number of oncogenic gene alterations, showed no clinical response to EGFR-TKI therapy. This observation supports the concept that it is not just major driver mutations that affect therapeutic response and clinical outcome, and highlights the importance of conducting comprehensive genome sequencing in patients with lung adenocarcinoma.

In regard to the *EGFR* gene, we identified differences in DFS and OS between patients with *EGFR* active mutation and those with wildtype *EGFR* or non-active *EGFR* mutations. Although the prognostic impact of *EGFR* mutation in lung adenocarcinoma remains controversial, several retrospective studies have reported that patients with *EGFR* mutation survived for longer periods than those without mutations, irrespective of therapy^25,26^. Such results may in part be a reflection of cohort composition in regard to gender and smoker status, with several investigators having reported that among patients with NSCLC, those that have never smoked have a better OS than smokers^24,27^.

It is important to point out that the current study has some limitations. Firstly, this is a retrospective study with a relatively small number of patients. Secondly, we noted an imbalance between the proportion of Stage I/II and Stage III/IV surgically resected cases in comparison with reported data for other Japanese cohorts^28^. Since only patients from whom sufficient amounts of tumor DNA could be extracted from FFPE specimens were included in this study, the proportion of advanced stage cases in our study was understandably higher. Third, when assessing the impact of each genomic alteration on DFS and OS, we did not perform multiple testing correction. This is because we analyzed only about 10 genomic alterations on survival and the sample size of this study was limited due to the cost of NGS analyses. However, to our knowledge, the present study represents the largest cohort of Japanese lung adenocarcinoma patients to have been genomically characterized using a comprehensive gene panel, and thus contributes substantially to our understanding of the clinical progression of lung adenocarcinoma in this population.

In conclusion, using an NGS sequencing approach we have identified actionable genomic driver mutations in 98% of Japanese lung adenocarcinoma patients, and discovered that the mutation frequencies of several key genes in this population appear to differ from those described in the TCGA database. Notably, concurrent loss of *CDKN2B* or *RB1* was associated with poor prognosis in patients with *EGFR* active mutation. In addition, our data indicate that lung adenocarcinoma patients with high numbers of oncogenic gene alterations may show the worst responses to EGFR-TKI targeted therapy. Although further studies are needed to verify these findings, our data improve our understanding of the relationship between genomic alteration and prognosis in lung adenocarcinoma.

## Material and Methods

### Patients and tissues

This study was approved by the Institutional Review Boards of Niigata University, and Kyusyu University Hospital. At Niigata University, patients were recruited from January 2008 to December 2014, and at Kyushu University from October 2013 to August 2015. All methods were performed in accordance with the relevant guidelines and regulations, and written informed consent was obtained from all patients. Patients were selected using the following three criteria: firstly, a tumor content of >20% based on pathological review of hematoxylin and eosin (H&E) stained slides. Secondly, radiological confirmation of lung adenocarcinoma with a consolidation/tumor ratio (C/T ratio) >0.5 using thin-section computed tomography (CT). Thirdly, the successful extraction of ≥150 ng DNA from each sample. One hundred patients who underwent surgery for primary lung adenocarcinoma were finally enrolled into the study, with all clinicopathology data, including smoking history, being retrieved from medical records. Six patients with Stage IV disease were included in our cohort. Preoperative work up revealed no metastasis and their diseases were clinically diagnosed as Stage I in 3 patients, Stage II in 2 patients, and Stage III in one preoperatively, however, they were found to have dissemination to visceral pleura by postoperative pathological examination (Table 1).

### Sequencing library preparation

Formalin-fixed, paraffin embedded (FFPE) cancer tissue from surgical specimens was used for analysis. An independent pathologist evaluated the tumor content using H&E slides. Where applicable, unstained slides were macro-dissected to enrich for tumor content and genomic DNA was extracted using the BiOstic FFPE Tissue DNA Isolation Kit (Mo Bio Laboratories, Inc; Carlsbad, CA). Sample preparation, genomic sequencing and subsequent analyses were all performed in a CLIA/CAP-accredited laboratory (KEW Inc.; Cambridge, MA).

### Comprehensive genomic sequencing

FFPE genomic DNA (150 ng) was converted into libraries and enriched for a 415 gene panel with CANCERPLEX (KEW Inc.; Cambridge, MA). CANCERPLEX is a clinically validated gene panel enriched for the coding regions and selected introns of 415 genes with known relevance for cancer. Sequencing was performed on the Illumina MiSeq and NextSeq platforms with an average of 500× sequencing depth. Genomic data were then processed through a proprietary bioinformatics platform and knowledge base to identify multiple classes of genomic abnormalities, including single-nucleotide substitutions (SNPs), small insertions/deletions (indels), copy number variations (CNVs), and translocations in *ALK*, *RET*, and *ROS1*. A threshold of 10% allelic fraction was used for SNPs and indels, and thresholds of >2.5-fold (gains) and 0.5-fold (losses) were used for CNVs. To assess the somatic status of mutations in the absence of constitutive samples, we employed a filtering strategy similar to one recently published, but with minor differences^29,30^. Based on both published evidence and our own experience, this approach allows the correct discrimination between germline and somatic variants in >99% of cases. Mutation burden was determined by the number of nonsynonymous SNPs present in the tumor that had population frequencies of <1% in dbSNP and the 1000 genomes databases. Actionable mutations were defined as known oncogenic alterations in key driver genes that are associated with response to approved targeted therapies (e.g., exon 19 deletions in *EGFR*). The number of genomic alterations was calculated in the context of the total number of genes represented on the CANCERPLEX panel. *EGFR* active mutations were defined as those involving *EGFR* exon 19 deletion or an L858R point mutation.

### Statistical analysis

Associations between each genotype and clinical characteristics were analyzed using two-tailed Student’s *t* tests and Fisher’s exact tests. Disease-free survival (DFS) was defined as the time from surgery to documented clinical progression or death. Overall survival (OS) was defined as the time from surgery until death. Progression-free survival (PFS) was defined as the time from the treatment to disease progression or death from any cause. As for PFS, we have established the cutoff value (4 or more vs. less than 4), which was based on the median number of identified genomic alterations in all patients. Survival curves were constructed using the Kaplan-Meier method, with statistical significance determined by log-rank test. Univariate and multivariate Cox proportional hazards models were developed to determine which factors had a significant impact on survival and to assess independent prognostic significance. All factors that attained a significance level of *p* < 0.05 in univariate analysis were included in the multivariate analysis. Statistical analysis was performed using SPSS for Windows Version 23.0 (SPSS, Inc., Chicago, IL, USA), with *p* < 0.05 being considered statistically significant.

## Electronic supplementary material

Supplementary Table S1, Table S2, Table S3, Table S4, Table S5, and Figure S6
