# Supplementary material for: Impact of Concurrent Genomic Alterations Detected by Comprehensive Genomic Sequencing on Clinical Outcomes in East-Asian Patients with EGFR-Mutated Lung Adenocarcinoma
Source: Sci Rep. 2018 Jan 17;8:1005. doi: 10.1038/s41598-017-18560-y (PMC5772517; doi:10.1038/s41598-017-18560-y)
Supplement: Supplementary file 1 — Supplementary Table S1, Table S2, Table S3, Table S4, Table S5, and Figure S6 [file 41598_2017_18560_MOESM1_ESM.doc]

**Supplementary materials**

**Title**

Impact of Concurrent Genomic Alterations Detected by Comprehensive Genomic Sequencing on Clinical Outcomes in East-Asian Patients with EGFR-Mutated Lung Adenocarcinoma

**Author list**

Seijiro Sato

Masayuki Nagahashi

Terumoto Koike

Hiroshi Ichikawa

Yoshifumi Shimada

Satoshi Watanabe

Toshiaki Kikuchi

Kazuki Takada

Ryota Nakanishi

Eiji Oki

Tatsuro Okamoto

Kouhei Akazawa

Stephen Lyle

Yiwei Ling

Kazuaki TakabeShujiro Okuda

Toshifumi Wakai^*^

Masanori Tsuchida^*^

| **Supplementary Table S1. *EGFR-*Mutated Patient Demographics** | | |
| --- | --- | --- |
| Factor | Category | N = 48 |
| Age (Years) | Median Range | 66 39-84 |
| Gender (N) | Male | 25 |
|  | Female | 23 |
| Smoking (N) | PY <30 | 40 |
|  | PY ≥30 | 8 |
| *EGFR* mutation subtypes* (N) | Exon 19 deletion | 23 |
|  | Exon 21 (L858R) | 20 |
|  | Exon 18 (E709K) | 1 |
|  | Exon 20 (T790M) | 1 |
|  | Amplification | 1 |
|  | Others | 9 |
| Pathological stage (N) | I | 20 |
|  | II | 5 |
|  | III | 20 |
|  | IV | 3 |
| **Some patients had more than one EGFR genomic alteration;* | | |
| *N, number; PY, pack-years.* |  |  |

| **Supplemental Table S2. Distribution of Genomic Alterations in the EGFR-Mutated Patients (N = 48)** | |
| --- | --- |
| Genetic alterations identified | N (%) |
| Exon19 deletion | 20 (41.7) |
| L858R | 17 (35.4) |
| Exon19 deletion + T790M | 1 (2.1) |
| Exon19 deletion + A750P | 1 (2.1) |
| Exon19 deletion + I759L | 1 (2.1) |
| L858R + E709K | 1 (2.1) |
| L858R + L62R | 1 (2.1) |
| L858R + D262Y | 1 (2.1) |
| Amplification + A767del | 1 (2.1) |
| L62R | 1 (2.1) |
| S246N | 1 (2.1) |
| S306L | 1 (2.1) |
| Y1016H | 1 (2.1) |
| *N, number.* |  |

| **Supplementary Table S3. The Difference of Genomic Alterations Between the Patients with *EGFR* Active Mutation and the Patients with *EGFR* Wild Type or *EGFR* Non-active Mutation.** | | | | |
| --- | --- | --- | --- | --- |
|  |  | Patients with *EGFR* Active Mutations | Patients with *EGFR* Wild Type or *EGFR* Non-active Mutations |  |
|  |  |  |
| Factor | Category | N = 43 (%) | N = 57 (%) | *p* Value |
| *TP53* | WT | 31 (72.1) | 29 (50.9) | **0.026** |
|  | MUT | 12 (27.9) | 28 (49.1) |  |
|  |  |  |  |  |
| *CDKN2B* | WT | 27 (62.8) | 41 (71.9) | 0.225 |
|  | MUT | 16 (37.2) | 16 (28.1) |  |
|  |  |  |  |  |
| *RB1* | WT | 36 (83.7) | 43 (75.4) | 0.225 |
|  | MUT | 7 (16.3) | 14 (24.6) |  |
|  |  |  |  |  |
| *CDKN1B* | WT | 34 (79.1) | 47 (82.5) | 0.430 |
|  | MUT | 9 (20.9) | 10 (17.5) |  |
|  |  |  |  |  |
| *CDKN2A* | WT | 33 (76.7) | 50 (87.7) | 0.120 |
|  | MUT | 10 (23.3) | 7 (12.3) |  |
|  |  |  |  |  |
| *PTEN* | WT | 40 (93.0) | 43 (75.4) | **0.018** |
|  | MUT | 3 (7.0) | 14 (24.6) |  |
|  |  |  |  |  |
| *ARID1A* | WT | 35 (81.4) | 48 (84.2) | 0.456 |
|  | MUT | 8 (18.6) | 9 (15.8) |  |
|  |  |  |  |  |
| *APC* | WT | 39 (90.7) | 45 (78.9) | 0.093 |
|  | MUT | 4 (9.3) | 12 (21.1) |  |
|  |  |  |  |  |
| *STK11* | WT | 36 (83.7) | 49 (86.0) | 0.485 |
|  | MUT | 7 (16.3) | 8 (14.0) |  |
|  |  |  |  |  |
| *FBXW7* | WT | 37 (86.0) | 50 (87.7) | 0.517 |
|  | MUT | 6 (14.0) | 7 (12.3) |  |
|  |  |  |  |  |
| *KRAS* | WT | 43 (100) | 45 (78.9) | **<0.001** |
|  | MUT | 0 | 12 (21.1) |  |
|  |  |  |  |  |
| *ACVR2A* | WT | 38 (88.4) | 50 (87.7) | 0.588 |
|  | MUT | 5 (11.6) | 7 (12.3) |  |
| *Note: Only genes which were mutated in more than twelve patients were analyzed.* | | | | |
| *N, number; WT, wild type; MUT, mutation.* | | | | |
| *Bold values are those with statistical significance of p<0.05.* | | | |  |

| **Supplementary Table S4. Univariate Analysis of OS in Patients with EGFR Active Mutations** | | | | | |
| --- | --- | --- | --- | --- | --- |
|  |  |  |  | Univariate Analysis | |
| Genes | Category | N = 43 | 5-year OS (%) | HR (95% CI) | p-value |
| *CDKN2B* | WT | 27 | 83.3 |  |  |
|  | MUT | 16 | 70.7 | 5.099 (0.568–45.769) | 0.146 |
|  |  |  |  |  |  |
| *CDKN2A* | WT | 33 | 80.4 |  |  |
|  | MUT | 10 | 68.6 | 3.528 (0.578–21.524) | 0.172 |
|  |  |  |  |  |  |
| *CDKN1B* | WT | 34 | 71.1 | 41.312 (0.033–51926.332) | 0.307 |
|  | MUT | 9 | 100 |  |  |
|  |  |  |  |  |  |
| *TP53* | WT | 31 | 79.9 |  |  |
|  | MUT | 12 | 75.0 | 0.998 (0.181–5.508) | 0.998 |
|  |  |  |  |  |  |
| *STK11* | WT | 36 | 76.2 | 27.271 (0.001–500395.607) | 0.509 |
|  | MUT | 7 | 100 |  |  |
|  |  |  |  |  |  |
| *RB1* | WT | 36 | 85.9 |  |  |
|  | MUT | 7 | 50.0 | 13.442 (1.203–150.148) | **0.035** |
|  |  |  |  |  |  |
| *ARID1A* | WT | 35 | 74.9 |  |  |
|  | MUT | 8 | 100 | 0.619 (0.067–5.725) | 0.673 |
|  |  |  |  |  |  |
| *FBXW7* | WT | 37 | 79.5 |  |  |
|  | MUT | 6 | 75.0 | 2.125 (0.346–13.040) | 0.416 |
|  |  |  |  |  |  |
| *EGFR** | WT | 37 | 69.4 |  |  |
|  | MUT | 6 | 80.0 | 1.527 (0.263–8.862) | 0.637 |
| *Note: Only genes which were mutated in more than six patients were analyzed* | | | | | |
| **Refers to non-active EGFR mutations i.e. excluding Exon19 deletion and L858R* | | | | |  |
| *OS, overall survival; N, number; HR, hazard ratio; CI, confidence interval; WT, wildtype; MUT, mutated;* | | | | | |
| *Bold values are those with a statistical significance of p<0.05.* | | | | |  |

| **Supplementary Table S5. Univariate Analysis of OS in Patients with Wildtype *EGFR* or Non-active EGFR Mutations** | | | | | |
| --- | --- | --- | --- | --- | --- |
|  |  |  |  | Univariate Analysis | |
| Genes | Category | N = 57 | 5-year OS (%) | HR (95% CI) | p-value |
| *TP53* | WT | 29 | 72.7 |  |  |
|  | MUT | 28 | 42.7 | 2.025 (0.807–5.081) | 0.133 |
|  |  |  |  |  |  |
| *CDKN2B* | WT | 41 | 50.2 |  |  |
|  | MUT | 16 | 69.6 | 0.419 (0.122–1.433) | 0.166 |
|  |  |  |  |  |  |
| *RB1* | WT | 43 | 50.1 |  |  |
|  | MUT | 14 | 71.4 | 0.761 (0.254–2.281) | 0.626 |
|  |  |  |  |  |  |
| *PTEN* | WT | 43 | 49.7 |  |  |
|  | MUT | 14 | 68.8 | 0.675 (0.225–2.024) | 0.483 |
|  |  |  |  |  |  |
| *KRAS* | WT | 45 | 57.8 |  |  |
|  | MUT | 12 | 44.4 | 1.725 (0.660–4.507) | 0.266 |
|  |  |  |  |  |  |
| *APC* | WT | 45 | 53.7 |  |  |
|  | MUT | 12 | 55.6 | 1.155 (0.417–3.199) | 0.781 |
|  |  |  |  |  |  |
| *CDKN1B* | WT | 47 | 54.8 |  |  |
|  | MUT | 10 | 58.3 | 0.854 (0.284–2.566) | 0.778 |
|  |  |  |  |  |  |
| *ARID1A* | WT | 48 | 55.9 |  |  |
|  | MUT | 9 | 48.6 | 1.693 (0.563–5.096) | 0.349 |
|  |  |  |  |  |  |
| *STK11* | WT | 49 | 60.4 |  |  |
|  | MUT | 8 | 19.4 | 2.829 (1.018–7.862) | **0.046** |
|  |  |  |  |  |  |
| *BRCA2* | WT | 49 | 48.3 |  |  |
|  | MUT | 8 | 87.5 | 0.240 (0.032–1.807) | 0.166 |
| *Note: Only genes which were mutated in more than eight patients were analyzed.* | | | | | |
| *OS, overall survival; N, number; HR, hazard ratio; CI, confidence interval; WT, wildtype; MUT, mutated;* | | | | | |
| *Bold values are those with statistical significance of p<0.05.* | | | |  |  |

**
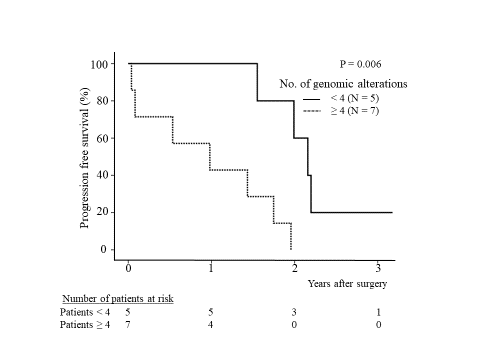
**

**Supplementary Figure S6.** Clinical response of *EGFR* mutated patients treated with EGFR tyrosine kinase inhibitor (EGFR-TKI). Progression-free survival curves for patients with or without 4 or more genomic alterations.
